# Severe Infections with Human Adenovirus 7d in 2 Adults in Family, Illinois, USA, 2014

**DOI:** 10.3201/eid2204.151403

**Published:** 2016-04

**Authors:** Adriana E. Kajon, Michael G. Ison

**Affiliations:** Lovelace Respiratory Research Institute, Albuquerque, New Mexico, USA (A.E. Kajon);; Northwestern University Feinberg School of Medicine, Chicago, Illinois, USA (M.G. Ison)

**Keywords:** adenovirus, adenovirus 7d, viruses, respiratory infections, family, Illinois, United States

## Abstract

Human adenovirus 7d, a genomic variant with no reported circulation in the United States, was isolated from 2 adults with severe respiratory infections in Illinois. Molecular typing identified a close relationship with strains of the same genome type isolated from cases of respiratory disease in several provinces of China since 2009.

Species B human adenoviruses are frequent causative agents of acute respiratory disease requiring hospitalization mainly for children but occasionally also for adults. In particular, respiratory infections with human adenovirus 7 (HAdV7) have a well-documented association with severe clinical manifestations, nosocomial transmission, prolonged hospitalizations, pulmonary sequela, and deaths (*1**−**6*). Adenovirus pneumonia among immunocompetent adults is still relatively infrequent and therefore attracts considerable attention (*4*).

In the absence of active sentinel surveillance for adenovirus infections, there is no current sense of what the actual burden of adenovirus-associated respiratory disease is in the United States or which are the most prevalent pathogenic types circulating in this country. Only large outbreaks or severe cases elicit enough interest to pursue etiology investigation. We report characterization of adenovirus strains isolated from 2 adults in the same family who had severe respiratory infections in Illinois, USA, during December 2014.

## The Study

This study was approved by the Northwestern University institutional review board. A waiver of consent was provided by this board.

Patient 1 was a 44-year-old man (electrician) who had no previous medical problems. He came to a hospital on December 2, 2014, with chest pain, fever (temperature up to 101°F), cough and shortness of breath, a few days after returning from a visit with family in Alabama. Two respiratory specimens were positive for adenovirus by PCR.

The patient was transferred to Northwestern Memorial Hospital (NMH) (Chicago, IL, USA) because of respiratory failure on December 13. A nasal swab specimen obtained at admission to NMH was positive for adenovirus B/E by the GenMarkDx eSensor Respiratory Virus Panel (Genmark Diagnostics, Inc., Carlsbad, CA, USA). He was given a diagnosis of adenovirus pneumonia, acute heart failure, and respiratory failure that required veno−venous extracorporeal membrane oxygenation during December 14−19. He slowly recovered but remained hospitalized until cardiac function normalized and was discharged on January 17, 2015. No other viral, bacterial or fungal pathogens were detected in any multiple blood, induced sputum, and bronchoalveolar lavage (BAL) specimen cultures performed during hospitalization. A BAL specimen obtained on December 17 was positive for adenovirus B/E by PCR. Sputum specimens were negative for *Mycoplasma* spp. and *Chlamydia*. spp.

Patient 2, the father of patient 1, was a retired 68-year-old man with a history of coronary artery disease, chronic obstructive pulmonary disease, chronic kidney disease, and diabetes. He came to a hospital on December 14, 2014, with fever, weakness, and shortness of breath. He was transferred to NMH because of respiratory failure, admitted to the intensive care unit, and required intubation and mechanical ventilation during December 16−21. A BAL specimen obtained on December 17 was positive for adenovirus B/E by the GenMarkDx eSensor Respiratory Virus Panel. No other pathogens were detected in this specimen. The patient had a nearly complete recovery and was discharged from the hospital on December 25.

**Figure 1:**
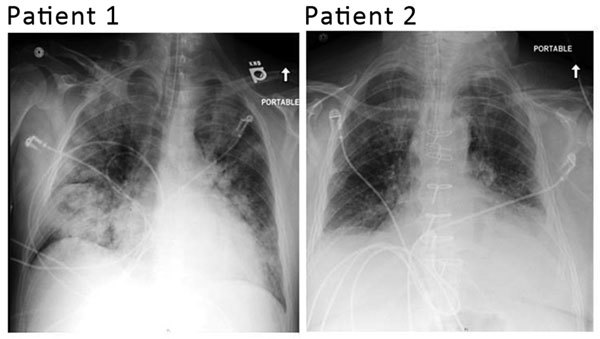
Admission chest radiographs of 2 adults with severe infections with adenovirus type 7 in family, Illinois, USA, 2014. Chest radiograph of patient 1 shows diffuse parenchymal consolidation involving all lobes. Chest radiograph of patient 2 shows interstitial changes in all lung fields and cardiomegaly.

**Figure 2:**
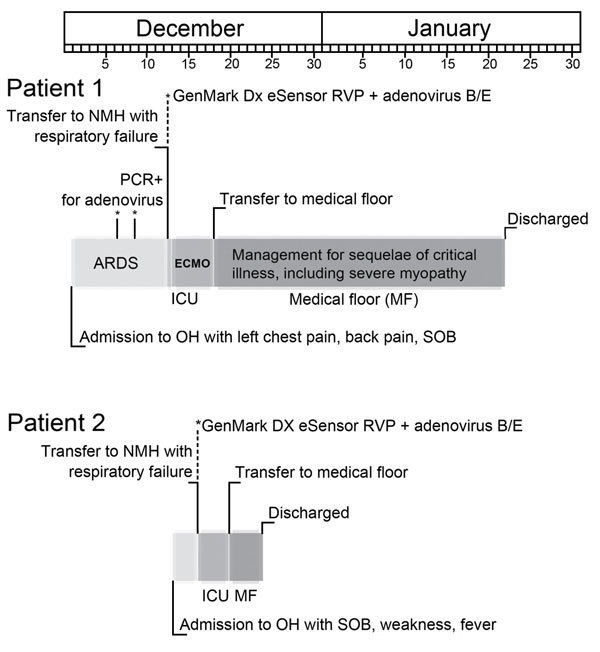
Timeline of events of 2 adults with severe infections with adenovirus type 7 in family, Illinois, USA, 2014. Shades of gray indicate different care units/wards in the hospital. RVP, respiratory virus panel; NMH, Northwestern Memorial Hospital (Chicago, IL, USA); ARDS, acute respiratory distress syndrome; ECMO, extracorporeal membrane oxygenation; ICU, intensive care unit; OH, outside hospital; SOB, shortness of breath.*Respiratory specimen positive for adenovirus by PCR.

No cases of respiratory illness were reported for other family members. Admission chest radiographs for both patients are shown in Figure 1, and a timeline of events are shown in Figure 2.

Adenovirus-positive clinical specimens (1 nasal swab specimen and 1 BAL specimen from patient 1 and 1 BAL specimen from patient 2) were shipped from NMH to Lovelace Respiratory Research Institute (Albuquerque, NM, USA) for molecular typing of the detected virus. HAdV was readily isolated in A549 cells from the nasal swab specimen obtained on December 13 at admission of patient 1at NMH, and from the initial culture of the BAL specimen obtained from patient 2 on December 17. These findings indicated that a high infectious virus load was present in the respiratory tract of both patients at the time of specimen collection.

Virus isolates from both patients were typed as HAdV7 by amplification and sequencing of the hexon gene hypervariable regions 1–7 and the complete fiber gene by using previously described primer pairs (*7*). Sequences of both amplicons (GenBank accession nos. KT266797−KT266800) were identical for the 2 isolates.

**Figure 3:**
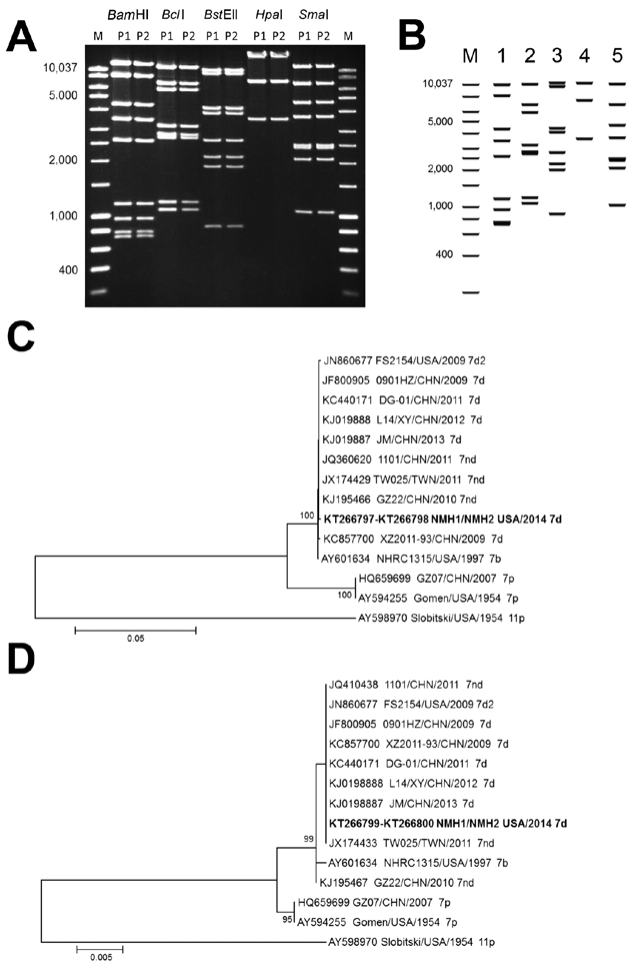
Enzyme and phylogenetic analysis of human adenovirus 7 isolates from 2 patients in Illinois, USA, 2014, and comparison isolates. A) Restriction enzyme analysis of genomic DNA from human adenovirus 7 isolates from patients 1 and 2 (P1 and P2, father and son). Lane M, molecular mass marker. Values on the left are in basepairs. B) Virtual restriction enzyme analysis of 6 complete genomic sequence of adenovirus strain DG01 from China. Lane m, Molecular mass marker; lane 1, *BamH*I; lane 2, *Bcl*I; lane 3, *BstE*II; lane 4, *Hpa*I; lane 5, *Sma*I. Values on the left are in basepairs. Phylogenetic analysis of C) hexon (HVR1–7) and D) fiber genes of adenovirus 7 strains from Asia and North America. Strains isolated in this study are indicated in bold. Phylogenetic trees were inferred by using the neighbor-joining method with 1,000 replicates. Bootstrap values are shown at branching points, and values <70% are not shown. Evolutionary distances were computed by using the Kimura 2-parameter method. There were 14 nt sequences and 1,509 positions in the final dataset for hexon gene analysis and 978 positions for fiber gene analysis. Analyses were conducted in MEGA6 (*10*). Hexon and fiber genes of human adenovirus 11p (strain Slobitski) were used as references. Scale bars indicate nucleotide substitutions per site.

DNAs extracted from infected A549 cells by described procedures (*8*) were examined by restriction enzyme analysis, which is a powerful molecular approach for discrimination of intratypic genetic variability (*9*). Both isolates showed identical restriction profiles for endonucleases *Bam*HI, *Bcl*I, *Bst*EII, *Hpa*I, and *Sma*I, which confirmed the epidemiologic link and determined their genome type as 7d (*9*). A comparison between these profiles (Figure 3, panel A) and the corresponding in silico−generated profiles for HAdV7 genomic sequences available from GenBank (accession nos. KC440171 [Figure 3, panel B], JF800905, JX625134, KC857700, and KJ019880−KJ019888), and phylogenetic analysis of hexon and fiber gene sequences performed by using MEGA version 6.0 software (*10*) (Figure 3, panels C, D) showed a close relationship of the strain from Illinois with strains described in association with cases of severe pneumonia recently reported in difference provinces in China (*11*) and in Taiwan (*12*).

## Conclusions

HAdV7 has been detected sporadically among cases of acute respiratory infection in the United States over the past 15 years, and genomic variants 7b, 7d2, and 7h have been identified among the isolated strains (*2*,*7*,*13**,*). Our molecular typing findings suggest that genome type 7d might be circulating in the United States as a recently introduced newly emerging variant probably imported from Southeast Asia where increased incidence of severe HAdV7-associated respiratory disease has been reported (*11*,*12*,*14*). The 2 patients in this study had not traveled outside the United States and had not had contact with persons who recently traveled to Asia.

Two clusters of cases of severe respiratory disease associated with detection of HAdV7 were recently identified in the northwestern United States and are currently under investigation at the Centers for Disease Control and Prevention (Atlanta, GA) (K.S. Scott et al., pers. comm.). Comparison of complete genomic sequences of isolated strains and genome typing data will be critical for investigation of possible epidemiologic links between these cases.

Although no adequate data are available to compare clinical presentations associated with infection by different HAdV7 genome types, differences in virulence and transmissibility are likely among them. This issue merits further investigation. Severe respiratory disease has been reported in association with various genomic variants of HAdV7 (*1*,*11*,*15*), which suggests that the severity of HAdV-associated disease might also be attributable to population or individual host differences in susceptibility. Although patient 1 did not have an unusual medical history, patient 2 had concurrent conditions that might have contributed to severity of disease.

The described cases provide another example of the fact that adenoviruses can cause severe influenza-like respiratory disease in immunocompetent adults, and together with numerous case reports, highlight the need for retaining testing for adenovirus infection in the differential diagnosis of the etiology of acute respiratory disease. Our study confirms the value of restriction enzyme analysis (conducted by using gel electrophoresis of digested DNA or in silico digestion of complete genomic sequences) as a powerful approach for discrimination of intratypic HAdV genetic variability for molecular epidemiology studies.

HAdV7-associated respiratory disease has been shown to be preventable by oral vaccination with a live nonattenuated virus formulation currently licensed for exclusive use among military personnel (http://www.cdc.gov/vaccines/hcp/vis/vis-statements/adenovirus.html). Licensing of this vaccine for use among civilians, or the design of next generation noninfectious vaccines, merits consideration for efficient intervention to prevent severe adenovirus-associated acute respiratory disease in the general population.

This report describes 2 cases of severe respiratory disease in adults associated with the apparent recent emergence of genome type 7d in the United States in 2014. In the possible scenario of a dispersion of this strain as a prevalent causative agent of acute respiratory disease requiring hospitalization, genome typing will be a valuable asset to surveillance and outbreak investigation efforts.
